# Curious Case of Facial Pain Mimicking Temporomandibular Disorder

**DOI:** 10.7759/cureus.65040

**Published:** 2024-07-21

**Authors:** Arunkumar KV, Kajal Awana, Shubham Sharma, Vidya Iyer, Amit Gupta

**Affiliations:** 1 Oral and Maxillofacial Surgery, I.T.S. Centre for Dental Studies and Research, Ghaziabad, IND

**Keywords:** temporomandibular disorders, facial pain, parasitic infection, lateral pterygoid muscle, cysticercosis

## Abstract

Facial pain is a common but complex complaint, frequently associated with dental issues or temporomandibular disorder (TMD). However, rare aetiologies can complicate conventional diagnoses and treatment approaches. We present a case of a 36-year-old male with persistent jaw pain and restricted mandibular movement, initially managed as a typical TMD case. Conventional treatments yielded no improvement, prompting advanced imaging, which identified an unusual mass within the lateral pterygoid muscle. A surgical excision of the mass was performed, and histopathological examination revealed a rare and unexpected diagnosis. This case highlights the importance of considering uncommon conditions in the differential diagnosis of TMD and facial pain to ensure timely and appropriate therapeutic interventions.

## Introduction

Facial pain is a common but complex complaint, often linked to dental issues or temporomandibular disorder (TMD). However, rare causes can challenge conventional diagnoses. This case report outlines a patient's journey from tooth pain to the discovery of an uncommon underlying condition.

Initially, the pain was attributed to dental pathology, leading to a tooth extraction. When symptoms persisted, a provisional diagnosis of TMD was made. Advanced imaging eventually revealed an unexpected cause, emphasising the need for a broad differential diagnosis and comprehensive evaluation in the maxillofacial region.

## Case presentation

A 36-year-old male initially sought dental care for persistent tooth pain in the right maxillary posterior region at a nearby dental clinic, one month before reporting to our department. Clinical examination of the right maxillary third molar revealed grade 2 mobility, leading to the decision to extract the tooth. The procedure was routine except for a reduced mouth opening of 25 mm, with the normal mouth opening for an adult being 40-45 mm. Post-extraction, the patient developed a dry socket, causing severe pain and delayed healing, which was managed with regular dressings and analgesics. Despite these interventions, the patient's discomfort persisted, leading to a cascade of consultations and treatments to address the lingering facial pain.

The journey commenced with a referral to the medicine department for a comprehensive evaluation. A thorough examination was conducted, including an assessment of TMJ function and musculoskeletal integrity. No classical signs of TMJ dysfunction, such as clicking or popping, were noted except for decreased mouth opening, pain, and tenderness upon opening and closing the mouth, leading to a provisional diagnosis of TMD. Conservative management was initiated, including muscle relaxants, analgesics, alternating hot and cold fomentation, and daily ultrasound therapy for seven days. However, following the ultrasound therapy sessions, the patient reported a significant increase in pain severity.

**Figure 1:**
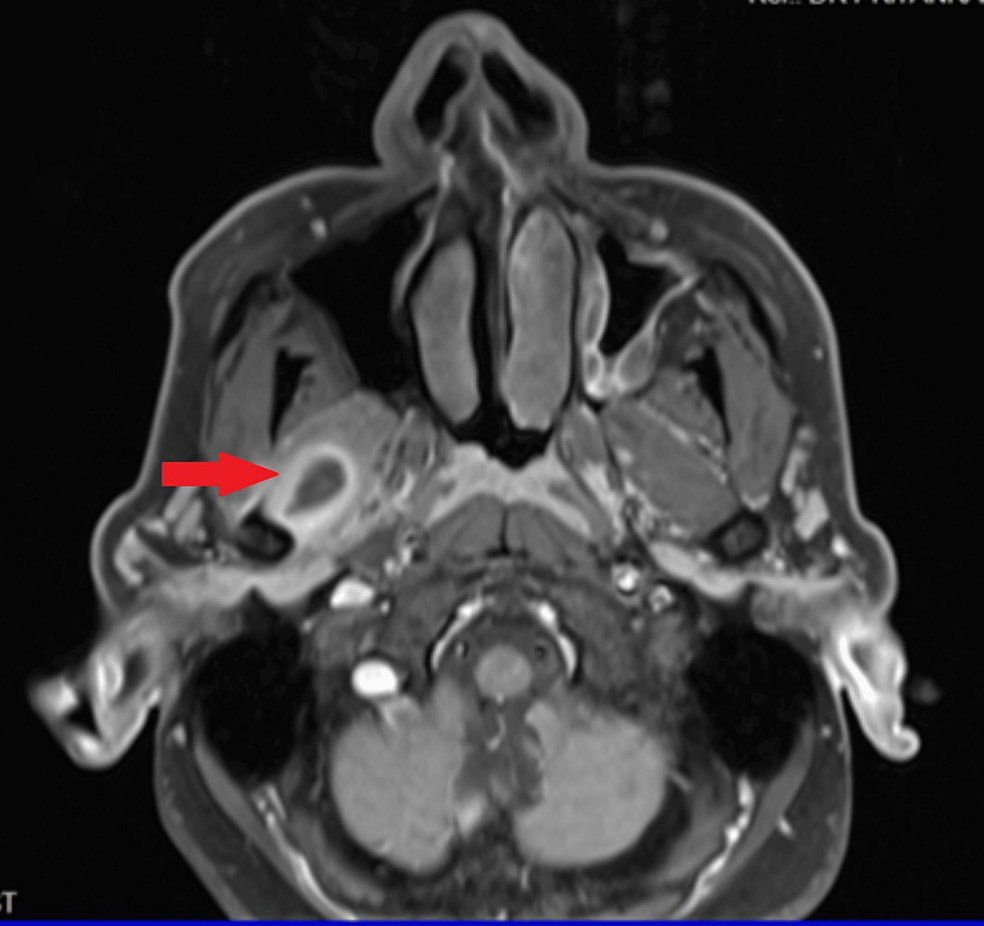
The axial section of the MRI shows a ring-enhancing lesion within the left lateral pterygoid muscle, suggestive of cysticercosis.

As symptoms worsened, the patient consulted an ENT specialist, yielding no notable issues upon evaluation. The patient was then referred to a neurologist, and an MRI of the craniofacial region was ordered. The MRI revealed a cystic lesion in the right lateral pterygoid muscle with a peripheral rim and surrounding oedema, suggestive of possible cysticercosis (Figure 1). The neurologist prescribed medication for the parasitic infection. Despite adhering to the prescribed regimen, the patient reported persistent facial pain and limited mouth opening.

Given the lack of improvement in symptoms, the patient sought further evaluation and management at the Department of Oral and Maxillofacial Surgery at the I.T.S. Centre for Dental Studies and Research, Ghaziabad, Uttar Pradesh, India. The patient presented with complaints of persistent facial pain localised to the right preauricular region. The pain was described as severe and stabbing in nature, radiating to the temporal, maxillary, and mandibular regions on the right side, and accompanied by difficulty in opening the mouth fully. The onset of symptoms was insidious, with no identifiable triggering event reported, though temporary relief was experienced with analgesics. Palpatory findings were non-contributory. The patient's mouth opening gradually decreased, reaching a limited opening of 15 mm by the time of presentation. Associated paraesthesia of the lower lip, chin, and half of the tongue on the right side was noted. Additionally, sensitivity in the lower right anterior teeth, spanning from the right mandibular central incisor to the right mandibular second premolar, was noted.

Analgesics were of no help; the patient's discomfort and pain progressed from the lower lip and chin to the right temples, upper jaw, and cheek, suggesting possible neural involvement or a secondary complication.

The patient was diabetic under treatment with oral hypoglycemic agents and had a history of tuberculosis treated 10 years ago with no current symptoms. Following an extensive review of the patient's medical history and diagnostic tests, a preliminary diagnosis of cysticercosis involving the right pterygoid was made.

**Figure 2:**
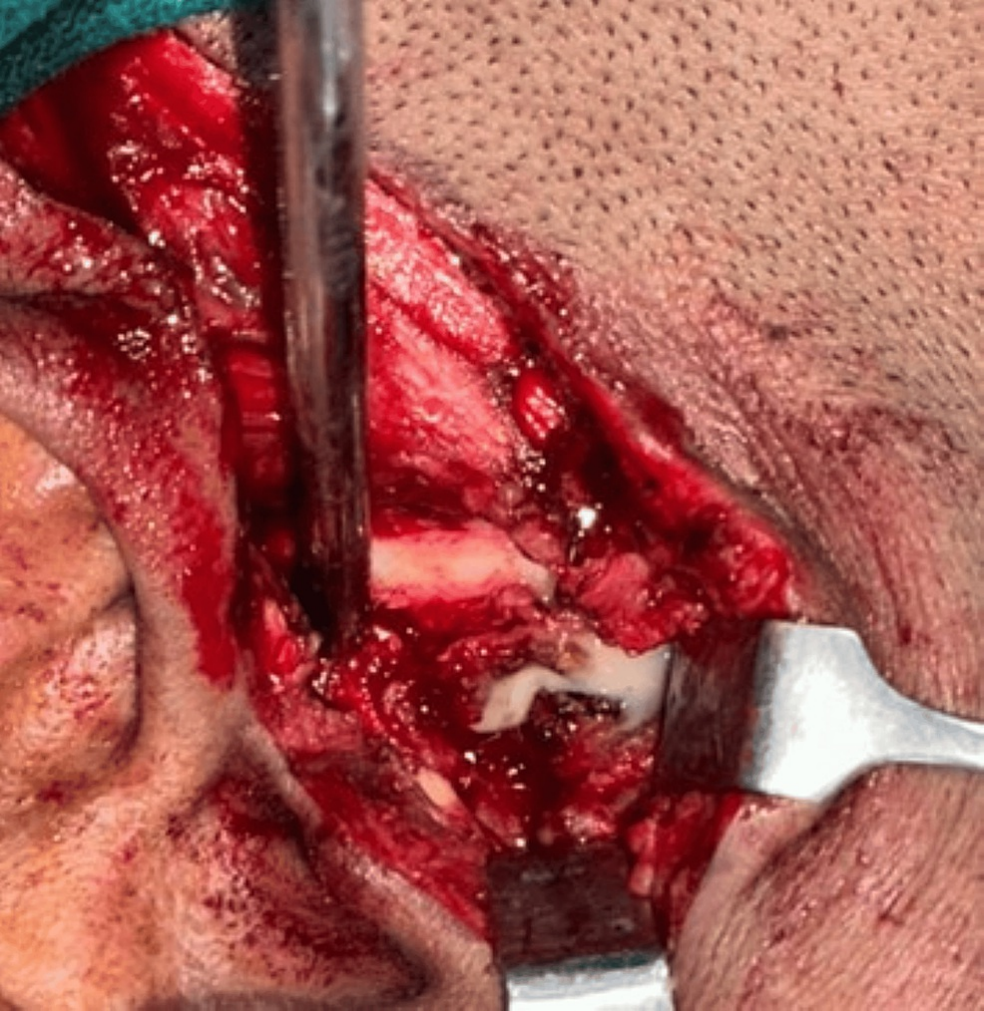
Frank pus-like material is seen in the space surrounding the ramus of the mandible.

Initially, conservative management with albendazole 400 mg thrice a day was attempted, but the patient returned after two days with unbearable pain. Due to persistent pain, the decision was made to proceed with surgical exploration. An excisional biopsy via condylar access osteotomy under general anaesthesia was performed. An extended preauricular incision was made to expose the condylar head. Osteotomy cuts were made at the condylar neck, and the condylar head was then osteotomized out of the joint space and preserved. Upon entry into the space surrounding the ramus, significant pus was encountered, which was drained and sent for culture and serum evaluation (Figure 2).

**Figure 3:**
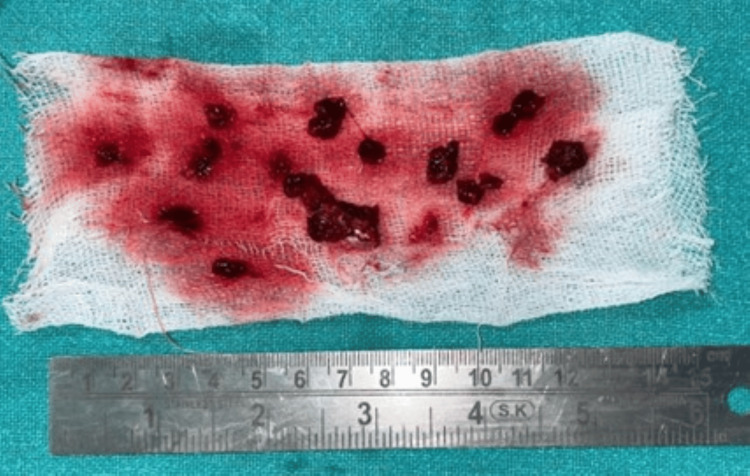
Cystic lining along with the debrided necrotic tissue

The cystic lining was removed, and any necrotic tissues were debrided and sent for histopathological examination (Figure 3). Thorough irrigation was performed with hydrogen peroxide, followed by 2% povidone-iodine and 0.9% normal saline. The preserved condylar head was then fixed using a 2.5-mm four-hole continuous plate. Extracorporeal fixation was done for the condylar head, which was then fixed to the ramus. Once haemostasis was achieved, the incision was closed in layers with a suction drain in place.

The pus culture did not reveal any microbial growth, with the enzyme-linked immunosorbent assay (ELISA) revealing an increase in the IgG levels. Additionally, the histopathological examination confirmed that the lesion was a cysticercus cyst.

Postoperatively, the patient was prescribed a course of albendazole to eradicate any remaining parasites. Follow-up examinations were conducted at one and three months post surgery. The patient reported no symptoms and exhibited good healing of the surgical site.

At the three-month follow-up, the patient remained asymptomatic with no signs of recurrence or complications. The prognosis in cases of oral cysticercosis treated with complete surgical excision is generally excellent, as the lesion is fully removed and the risk of recurrence is minimal, especially with adjunctive antiparasitic therapy.

## Discussion

Cysticercosis is caused by the larval stage of the tapeworm, *Taenia solium* (*T. solium*). The brain, skeletal muscle, and eye are the most commonly affected sites. Neurocysticercosis (involving the brain) is a potentially life-threatening parasitic infection. Cysticercosis in the muscles rarely affects the orofacial region in humans. The larvae, or cysticerci, inhabit the muscles and tissues of pigs, the intermediate hosts. Humans become infected by consuming undercooked pork containing cysticerci or by inadvertently ingesting *T. solium* eggs [1, 2]. Humans contract cysticercosis through faecal-oral contamination with *T. solium *eggs. Even vegetarians can acquire it by ingesting contaminated food or water or touching surfaces contaminated with faecal matter containing the eggs. Thus, pork consumption is a common, but not exclusive, transmission route [3]. In this particular instance, the acquisition of cysticercosis in a vegetarian individual could be attributed to the ingestion of raw, contaminated food. There's also a proposed mechanism where proglottids are regurgitated and re-ingested, potentially contributing to infection in individuals with taeniasis [4]. Gastric acid and intestinal fluids release invasive oncospheres from the eggs, which penetrate the bowel wall, enter the bloodstream, and travel to tissues like muscles. They settle in small blood vessels, forming cysts called cysticerci that typically reach about 1 cm in size within two to three months [5,6]. Clinical symptoms of cysticercosis vary by infection site, cyst number, and the host's immune response, leading to diverse manifestations. There is no general gender predilection, though regional variations may exist. While commonly affecting those in their 30s and 40s, cases can occur from the first to sixth decades [7]. The tissues most frequently affected by cysticercosis are subcutaneous tissue, the brain, striated muscles of the neck, heart, liver, lungs, orbit, peritoneum, and meninges [8]. Cysticercosis can rarely affect oral and perioral tissues, including masticatory and facial muscles, the tongue, buccal mucosa, and lips. Isolated muscular cysticercosis in the head and neck without central nervous system (CNS) involvement is uncommon and often presents with nonspecific symptoms, posing a diagnostic challenge [9]. Cases of cysticercosis in the oral and maxillofacial regions are uncommon, despite the abundance of vasculature and muscular tissue in this area. Reported cases within the masticatory muscles have involved the masseter and temporalis muscles [7]. To the best of our knowledge, prior to this report, only two cases of cysticercosis in the lateral pterygoid muscle had been documented [7, 10], excluding the non-English literature.

Clinical features of the disease vary depending on the infection site. Temporalis muscle involvement may present as temporal pain, headaches, temple swelling, and difficulties in mastication and speech [11-14]. Only two cases of cysticercosis of the temporalis presented with swelling, pain, and subsequent limitation of mouth opening [12]. Cysticercosis involving the masseter may present with pain and swelling in the mandible [13-15]. In the case involving the lateral pterygoid muscle, the patient presented with symptoms such as limited mouth opening, and spontaneous intense pain without a history of fever or headaches [7], which parallels our current case. Notably, in our case, the patient also reported paraesthesia in the lower lip and ipsilateral cheek.

Different muscular forms of cysticercosis exhibit distinct clinical manifestations. In the myalgic type, a focal mass-like behaviour is observed. The pseudotumor or abscess-like type often includes a necrotic component resembling an abscess. A relatively rare pseudohypertrophic form is characterised by muscle hypertrophy. Larval death leads to leakage of cystic fluid, inducing acute inflammation, which causes localised symptoms that vary based on cyst location [13]. In the current case, we identified the pseudotumor or abscess-like type of cysticercosis. During the exploration of the space around the lateral pterygoid/ramus, we encountered a frank pus-like discharge.

Diagnosing intramuscular cysticercosis is challenging due to nonspecific clinical manifestations, which can resemble conditions like lipoma, fibroma, neurofibroma, TMD, or intramuscular abscess. Plain radiography typically doesn't reveal active cysticerci but may show calcified lesions in chronic stages. Confirmation methods include high-resolution ultrasonography, ultrasound-guided fine-needle aspiration cytology, MRI, serum ELISA, and histopathology; MRI, considered a gold standard technique, is extensively used for diagnosing neurocysticercosis, as it can visualise the cyst along with the scolex [16]. In the case report by Kalladka et al., an MRI of the TMJ revealed a ring-enhancing lesion within the left pterygoid muscle, suggesting cysticercosis [7]. Similarly, in the current case, MRI revealed a cystic signal intensity lesion with an eccentric T2 hypointense focus within and peripheral post-contrast rim enhancement and surrounding oedema in the pterygoid muscles, indicative of cysticercosis with inflammation.

Other diagnostic methods for cysticercosis include limited faecal examination due to sporadic egg shedding and resemblance to eggs from other tapeworms. More accurate tests include enzyme-linked immunoblot transfer blot assay, Western blot for identifying *T. solium* antibodies, and monoclonal antibody HP10 assay, especially in cerebrospinal fluid [7]. In this case, elevated IgG antibody levels and histopathological examination confirmed cysticercosis.

Cysticercosis typically carries a favourable prognosis when managed conservatively. Pharmacological treatment with albendazole or praziquantel aims to reduce parasite burden by inducing cyst death. Albendazole's superior penetration into cerebrospinal fluid allows it to target subarachnoid and ventricular cysts. Combining it with corticosteroids enhances its anti-inflammatory efficacy [8]. Albendazole is commonly administered at doses of 15 mg/kg in adults [17]. The duration of treatment varies depending on the severity of the disease and may range from eight, 15, to 30 days [18]. In this case, albendazole was initially prescribed; however, due to the increased severity of the pain, surgery became necessary. The use of steroids remains controversial. Surgical excision is the preferred treatment, with a good prognosis and no recurrence in the maxillofacial region [2].

## Conclusions

This case highlights the diagnostic journey and management challenges encountered by a patient presenting with facial pain, paraesthesia, and dental sensitivities. Through interdisciplinary collaboration, cysticercosis in the pterygoid muscle was identified, despite its rarity. It underscores the importance of maintaining a broad differential diagnosis and using advanced imaging techniques. Sharing our experience aims to enhance awareness and timely management of cysticercosis and rare facial pain syndromes.
